# Personalized three-dimensional printed polyether-ether-ketone prosthesis for reconstruction after subtotal removal of chronic clavicle osteomyelitis: A case report

**DOI:** 10.1097/MD.0000000000025703

**Published:** 2021-04-30

**Authors:** Chang Chen, Yiran Yin, Huan Xu, Zhong Li, Fuyou Wang, Ge Chen

**Affiliations:** a Department of Orthopaedics, The Affiliated Hospital of Southwest Medical University; b Sichuan Province Laboratory of Orthopaedic Engineering, Luzhou City, Sichuan Province; c Center for Joint Surgery, Southwest Hospital, Third Military Medical University, Chongqing, China.

**Keywords:** bone defect, chronic clavicle osteomyelitis, clavicle reconstruction, PEEK, subtotal claviclectomy, three-dimensional printing

## Abstract

**Rationale:**

Three-dimensional (3D) printing has attracted wide attention for its potential and abilities in the assistance of surgical planning and the development of personalized prostheses. We herewith report a unique case of chronic clavicle osteomyelitis treated with a two-stage subtotal clavicle reconstruction using a 3D printed polyether-ether-ketone (PEEK) prosthesis.

**Patient concerns:**

A 23-year-old Chinese female presented to our clinic complaining about a progressive pain of her right clavicle for about 1 year.

**Diagnoses:**

Chronic clavicle osteomyelitis confirmed by percutaneous biopsy and lesion biopsy.

**Interventions:**

This patient accepted a long-term conservative treatment, which did not gain satisfactory outcomes. Thus, a subtotal removal and two-stage reconstruction of the right clavicle with a 3D-printed polyether-ether-ketone prosthesis stabilized by screw fixation system was performed.

**Outcomes:**

At 2-year follow-up, complete pain relief and satisfactory functional recovery of her right shoulder were observed.

**Lessons:**

Personalized 3D printed prosthesis is an effective and feasible method for reconstruction of complex bone defects.

## 1 Introduction

Reconstruction of complex bone defects after removal of an osteogenic tumor remains challenging in orthopedics, requiring bone transplantation and stable implant fixation. In recent years, three-dimensional (3D) printing technology has been widely used for various diseases.^[1,2]^ Because of the rapid prototyping and small-scale manufacturing based on digitized model and specific materials, 3D printing can make the production of objects easier, more accessible and affordable.^[3]^ In patients with a large bone defect after massive tissue removal, standard implants cannot provide a satisfactory reconstruction, while the individualized implants can be extremely helpful. With computer 3D remodeling based on individual data, patient-specific implant can be effectively fabricated.^[4,5]^

Patients with chronic clavicle osteomyelitis were rarely seen in clinical practice, and most of them were reported as a complication resulted from clavicle fracture or surgical procedure of head and neck.^[6,7]^ The infection usually occurs from hematogenous spread or trauma.^[8]^ The clinical manifestations of chronic clavicle osteomyelitis include pain, local redness and swelling, sometimes with thickened bone, rough surface and the formation of sinus.^[9]^ Diagnosis should only be confirmed by biopsy, suggesting the inflammatory lesions and without neoplasia.^[10]^ In most cases, antibiotics and other conservative treatment can attain a good outcome.^[11]^ However, when conservative treatments cannot provide a satisfactory clinical efficacy, the persistence of osteomyelitis can affect the function of shoulder joint and upper limb.^[12]^ Thus, if a long-term conservative treatment could not relief the symptoms, surgical procedure might be recommended.^[13]^

We herewith report a unique case of chronic clavicle osteomyelitis treated with a two-stage subtotal clavicle reconstruction using a 3D-printed polyether-ether-ketone (PEEK) prosthesis. This study was approved by the ethics committee of our hospital. The patient were informed and agreed to this study. Written consent was obtained from the patient.

## 2 Case presentation

A 23-year-old Chinese female patient presented to our clinic complaining about a 1-year history of a growing lump at the medial third of her right clavicle, associated with recurrent and aggravated pain. Limited range of motion of the right shoulder was reported as well. The patient experienced no compression and difficulty in breathing. Physical examination indicated the right shoulder had limitations in adduction, abduction, back extension, and elevation. A hard lump with a diameter of 7cm × 5 cm lump at the medial third of right clavicle was palpated with pain and slightly swelling. No redness and skin lesion around the lump; no muscular atrophy and weakness; no dislocation or deformity of the shoulder joint and no thoracic varicosity were observed.

**Figure 1:**
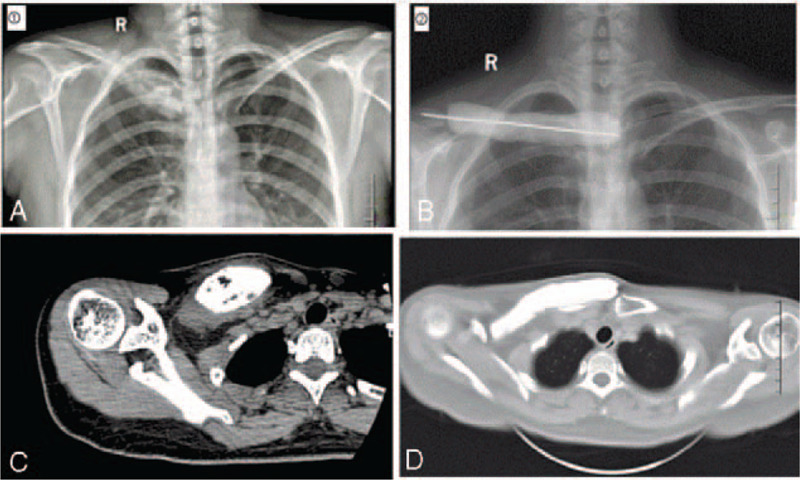
The X-ray and CT films of the patient: (1) at first admission; (2) after spacer implanted.

**Figure 2:**
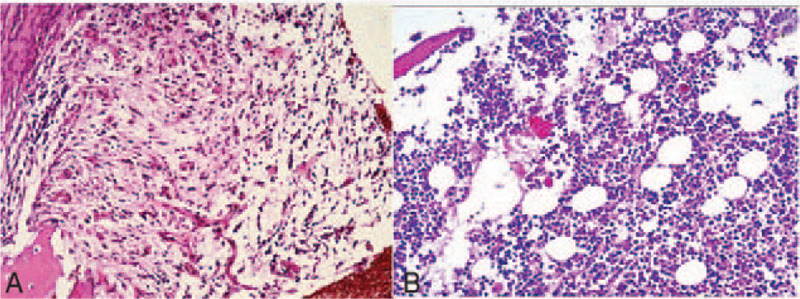
Pathological studies confirmed the diagnosis of chronic clavicle osteomyelitis: (A) Preoperative biology and (B) Second biology at the surgery of spacer implanted.

The X-ray film and computed tomography (CT) scan showed the sclerotic bone remodeling of the medial third of the right clavicle (Fig. 1). After admitted to the ward, a percutaneous bone biopsy was performed, revealing a suppurative inflammation with bone destruction, without the signs of malignancy (Fig. 2). A diagnosis of clavicle osteomyelitis was suggested. Thus, conservative treatments with antibiotic (based on drug sensitivity, clindamycin was given 1.2 g/d for 2 weeks) and non-steroidal anti-inflammatory drugs were given. The patient was discharged after the pain was relieved.

In the next 7 months, the symptom of pain gradually worsened and radiated to her right arm. Despite the intake of non-steroidal anti-inflammatory drugs and treatment with antibiotic, the pain was still obvious. Meanwhile, she started complaining of muscle weakness and limited mobility of the right shoulder. The lump of right clavicle was bigger than before and developing to her right neck. After admitted to the ward, X-ray film and 3D reconstruction of CT scan were carried out. Despite the conservative treatments were given, the pain and swelling of the right clavicle were still not relieved, seriously affecting the patient‘s life quality. Meanwhile, as a young woman, the growing lump of the right clavicle obvious affected the patient‘s appearance. Therefore, after the patient and her family members gave informed consent (Informed consent was obtained from the patient for publication of this case report details), we decided to perform a surgical procedure to remove the lesion and reconstruct the clavicle. Because the inflammatory lesion was limited in the medial third, a subtotal removal and two-stage reconstruction of the right clavicle was approved. In order to reconstruct the large structural defect after removal of the lesion without compromising the beauty of the neck, the application of a personalized 3D printed implant could be an ideal method.

**Figure 3:**
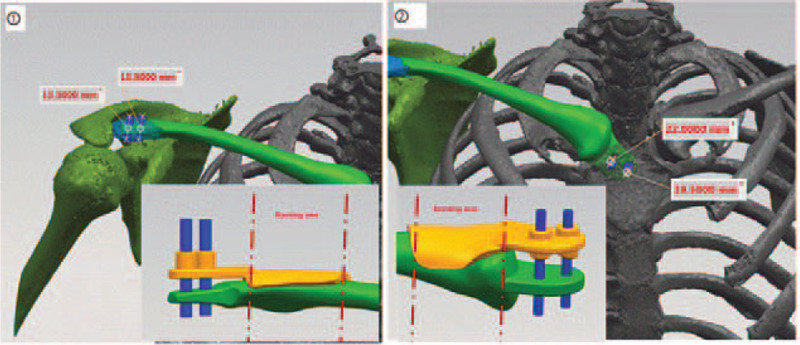
Designing and molding of the 3D implant used in the current case.

After complete removal of the lesion, the bone defect was replaced with a custom-made antibiotics-loaded (vancomycin) cement spacer (Fig. 3), which would allow the formation of an induced membrane according to the Masquelet technique.^[14]^ Meanwhile, a pathological biopsy was performed again, confirming the diagnosis of chronic osteomyelitis (Fig. 2). Six months later, a 3D-printed prosthesis could be implanted for the clavicle reconstruction.

The CT scan of chest and scapula were obtained by a scanner (Siemens) with 1.0 mm each layer. The data was stored and analyzed by Mimics 17.0 software in digital imaging and communications in medicine format. After the 3D model of the right clavicle was constructed by the Mimics 17.0 software (Mimics, Materialise, Leuven, Belgium), it was imported into SIEMENS NX software (Siemens PLM Software Inc, Germany) to design the guiding plate. The design of guiding plate was restored in stereolithography format and then printed by UP BOX+ 3D printer (Beijing Tiertime Technology Co, Ltd, China) using polylactic acid. 3D printing remodel based on the healthy contralateral left clavicle was conducted to simulate the prosthesis of right clavicle. In preoperative plan, the right clavicle was extended 15 mm laterally to ensure the same height with left shoulder (Fig. 3). The customized guide plates was installed to insert two screws into the acromial end of clavicle and two screws into the sternal stalk to ensure a strong fixation.

**Figure 4:**
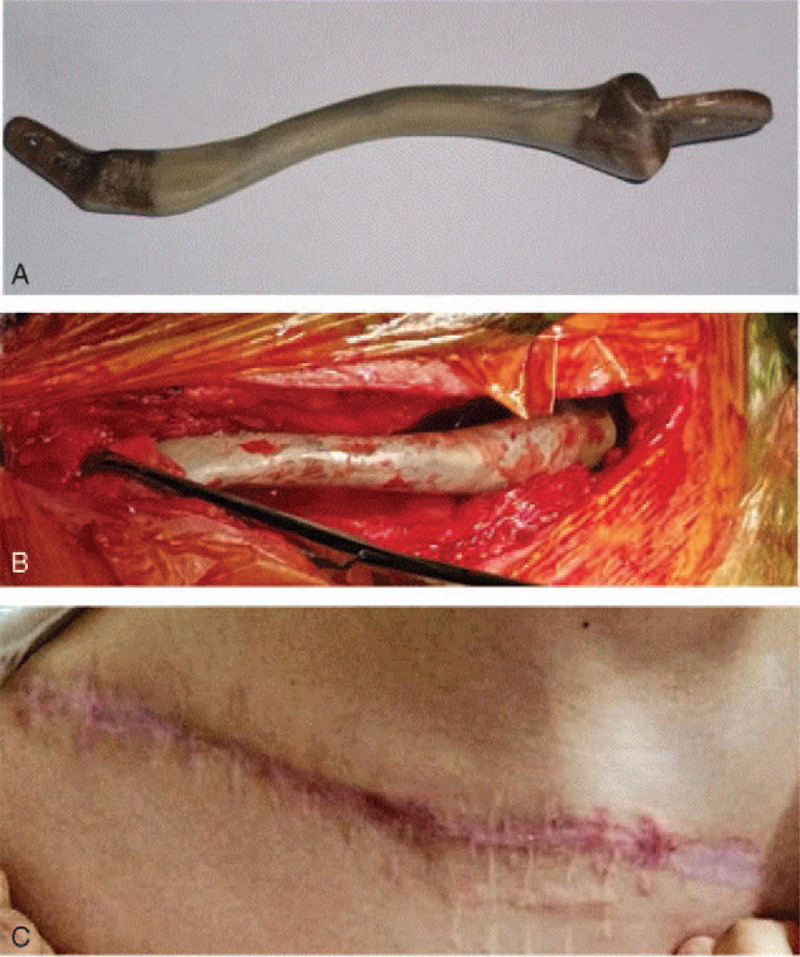
(A) The prosthesis used in the current case; (B) The surgical procedure of 3D implant fixation. (C) The surgical site healed without infection 3 months after operation.

The surgical procedure was carried out under general anesthesia, and the patient was positioned in a supine position. After the removal of spacer, the PEEK prosthesis was positioned in the Masquelet cavity. Two ends of the 3D-printed PEEK prosthesis was fixed into the acromial end and the medullary cavity of the sternal stalk respectively after cleaning the residual cavity (Fig. 4). The fracture of acromion end of clavicle was found during the operation, so it was fixed with steel wire. The blood vessels and nerves were carefully protected during the operation.

**Figure 5:**
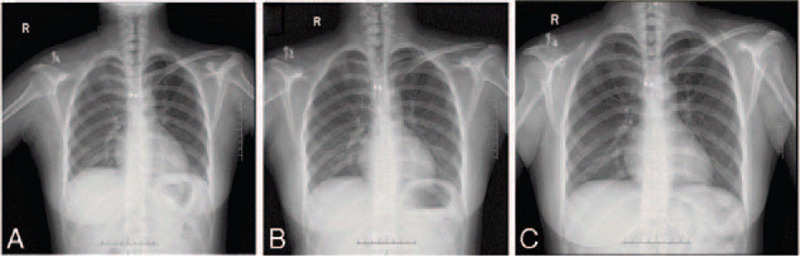
There was no failure or loosening of implant: (A) 1 month after operation; (B) 6 months after operation; (C) 1 year after operation.

**Figure 6:**
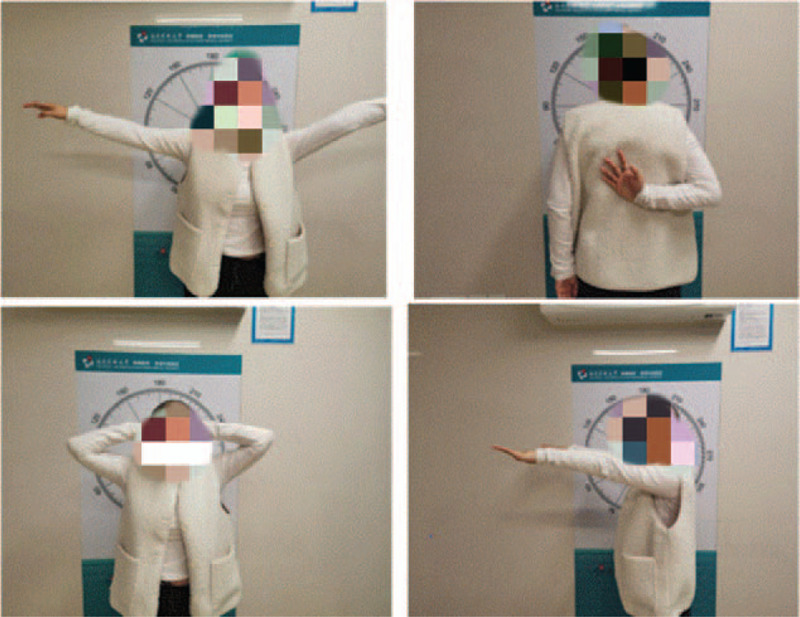
Recovery of should function 1 month after operation.

The patient achieved fast recovery after the surgery. Dressing replacement and removal of drainage occurs on post-operative day 1. The staples are removed on post-operative day 14. The functional exercise started ever since the drainage was removed. After 2-year follow-up, the patient indicated satisfactory cosmetic and functional outcomes. There was no failure or loosening of implant during 2-year follow-up (Fig. 5). The range of motion of right shoulder returned to the level of preoperative assessment 3 months after the surgical reconstruction (Fig. 6). While pain visual analogue score was 1/10 at 1 month and 0/10 at last 3 months. The evolution of the Constant-Murley shoulder score was satisfactory with an improvement to 88 points within 6 months. At the follow-up 2 years after operation, the patient reported a great satisfaction with the appearance and shoulder function.

## 3 Discussion

Chronic clavicle osteomyelitis was rarely reported in clinical practice. There was no consensus about the ideal treatment for chronic clavicle osteomyelitis. Early diagnosis and treatment was often missed, because most patients with chronic clavicle osteomyelitis were lack of pain or other typical symptoms.^[15]^ In most cases, the non-surgical treatment, including long-term antibiotics and non-steroidal anti-inflammatory drugs,^[6,7]^ can attain a satisfactory clinical outcome.^[16]^ Meanwhile, several studies suggested that when conservative treatment cannot work, surgical debridement and resection of the lesions were effective intervention for treating chronic nonbacterial osteomyelitis (CNO).^[17]^ Thaddeus et al reported a 10-year-old boy with CNO and was treated with a total clavicle removal. They suggested that the removal of an entire length of the clavicle was an effective and safe method for treating the CNO, without functional deficit and not sacrificing shoulder asymmetry.^[18]^ Patrick et al^[19]^ reported a patient who was diagnosed as a complicated CNO of the clavicle, and treated by a total clavicle reconstruction using free peroneal graft. Long-term follow-up showed a satisfactory outcome.

Whether reconstruction is needed after clavicular resection remains controversial. The clavicle provides attachment for muscles, offering support to body movement and increasing the mobility of shoulder by transferring the force from trapezius muscle to the scapula.^[20]^ Removal of the clavicle might result in weakened muscle strength and poor mobility of the shoulder joint. Meanwhile, the clavicle acts as a barrier protecting of neurovascular structures under the base of its medial third. The incidence of long-term complications, including infection and neurovascular injury, could increase if the protection were lost. Although some studies suggested that claviculectomy with and without reconstruction resulted in a similar short-term clinical outcome,^[21]^ a long-term research by Rubright et al^[22]^ suggested that patients with total clavicular resection would gradually lose some compensatory ability of functional deficit in long-term follow-up, as the clavicle contributes to the strength, coordinated scapulohumeral rhythm, and overall range of motion of the shoulder girdle.

In our case, considering that the claviclectomy might affect the long-term function of the shoulder and the symmetry of the body (this is what patients are most worried about.), the consensus of our surgeons was reconstruction of the clavicle. As the lesion was limited in medial third, we decided to save acromional end of clavicle and perform a two-stage subtotal clavicle reconstruction with a custom-made antibiotics-loaded cement spacer. The two-stage operation had two main advantages. First, we found that the infectious background was not suitable for a one-stage implantation. Meanwhile, we expected the cement spacer would contribute to the stability and biological integration of the 3D-printed prosthesis.^[14]^

Compared with free peroneal graft, 3D printing prosthesis can avoid the damage of the blood vessels and nerves of the lower extremities, which effectively reduced the operation time and blood loss. Meanwhile, in our case, after the subtotal clavicle resection, fixation of the free peroneal graft might be difficult, and postoperative complications such as nonunion might occur.

3D printing technology can precisely remodel the whole image of the bone defects and provide a personalized surgical planning.^[23]^ The personalized design of implants could also reduce the allogenic bone transplant, significantly reducing the time of operation.^[24,25]^ The initial CT data of contralateral clavicle was used as a reference for the design, allowing the implant keep in original shape of the right clavicle. Meanwhile, locking screws were used for distal prosthesis fixation, and the 3D printed guiding plate provided an accurate insertion of screws. Due to the anatomic variations and differences in bone defect, a more personalized implant with soft tissue docks can be beneficial for functional recovery of the patients. 3D printing can provide precise locations for the attachment of muscle and ligaments on the implants, increasing the stability of the implant and achieving a better functional recovery after surgery.^[26]^ It is an appropriate choice to apply 3D printing technology in complex orthopedic surgery.^[27]^ In our case, despite the partial removal of clavicle and surrounding soft tissues, the patient eventually achieved satisfactory mobility and function.

Compared to metal materials like titanium and tantalum, PEEK has been widely used for load-bearing orthopedic implant due to its good radiolucency on images and lower Young‘s modulus that can reduce stress-shielding.^[28,29]^ In our case, the excellent biological strength and smooth surface became the specific advantages of PEEK to reconstruct the clavicle. Carpenter et al^[30]^ suggested that the PEEK might have a poor osseointegration compared to roughened and porous surfaces. In our case, to ensure a stable reconstruction and fixation, we strengthened the prosthesis by lock screws at sternal stalk and acromional end respectively.

The patient reported a significant pain relief associated with a functional improvement at 2-year follow-up. To the best of our knowledge, the closest case report is the one by Goetti et al,^[19]^ in which a case of two-stage clavicular reconstruction using a free peroneal graft for a 21-year-old female patient presented with CNO of her left clavicle. They used a vascularized peroneal graft stabilized by ligamentous for the reconstruction. At 2-year follow-up, complete pain remission and improvement of the Constant-Murley score were reported, along with satisfactory cosmetic outcome. In addition, Liu et al^[29]^ retrospectively analyzed the tumor resection method used in 20 patients with clavicular tumors and evaluate its clinical efficacy. The average duration of follow-up care was 33.7 (12–71) months. Among five patients who underwent resection of malignant clavicular tumors and reconstruction, two of them underwent a re-operation because of a loose screw and plate displacement. In the functional assessment of the shoulder joint, patients with benign and malignant clavicular tumors showed significantly higher scores postoperatively compared with preoperative scores. For malignant clavicular tumors, no significant improvement was observed when comparing the non-reconstruction and reconstruction groups. The long-term outcome of this technique is not yet known. Finally, Lin et al suggested adoption of clavicle bone cement prosthesis for bone defect reconstruction after tumor resection can maintain the contour of shoulder and reduce the complications ascribe to the claviculectomy.^[31]^ In their study, the average Musculoskeletal Tumor Society score was 85.40% ± 5.68% (77%–90%), the mean visual analogue score was 1.40 ± 0.55 (1–2) and American Shoulder and Elbow Surgeons Shoulder Outcome Score was 92.40 ± 3.29 (87–96).

Although 3D printing technology has been a hot technology and was widely used around the world, it is still immature in China and needs plenty of research to guide its clinical application. This is also one of the important significance of our research. At present, several disadvantages of 3D printing limited its clinical utility. First, 3D printing needs specific equipment for designing and printing process, and most medical institutions did not have this condition. In this case, the designing and printing process took about 1 month. Meanwhile, additional ethical and technical regulations for 3D printing were still needed in our country. However, as the advantages of 3D printing far outweigh its disadvantages, and many technical problems have been solved with current studies, we do believe that 3D-printed implants will be widely used in the future and will benefit millions of patients.

## 4 Conclusion

Overall, our case report suggested that personalized 3D printed PEEK prosthesis is an effective method for the reconstruction of clavicle resection and other complex bone defects. We also suggested that the advantages and complications of clavicle reconstruction should be carefully discussed due to limited evidence of this pretty new technology. Meanwhile, in our case, the clinical outcomes of surgical reconstruction for treating chronic clavicle osteomyelitis were satisfactory in both functional and cosmetic aspects. However, it remains an isolated case and further reports are awaited to help surgeons and patients in their decision process.

## Acknowledgments

No benefits in any form have been received from a commercial party related directly or indirectly to the subject of this article. No funds were received in support of this study.

## Author contributions

**Conceptualization:** Chang Chen, Yiran Yin, Ge Chen.

**Data curation:** Chang Chen, Yiran Yin, Ge Chen.

**Formal analysis:** Chang Chen, Yiran Yin, Ge Chen.

**Funding acquisition:** Fuyou Wang, Ge Chen.

**Investigation:** Chang Chen, Yiran Yin, Ge Chen.

**Methodology:** Chang Chen, Ge Chen.

**Project administration:** Chang Chen, Ge Chen.

**Resources:** Chang Chen, Huan Xu, Ge Chen.

**Software:** Chang Chen, Huan Xu, Ge Chen.

**Supervision:** Chang Chen, Huan Xu, Ge Chen.

**Validation:** Chang Chen, Yiran Yin, Huan Xu, Ge Chen.

**Visualization:** Chang Chen, Yiran Yin, Ge Chen.

**Writing – original draft:** Chang Chen, Yiran Yin, Zhong Li, Fuyou Wang, Ge Chen.

**Writing – review & editing:** Chang Chen, Yiran Yin, Zhong Li, Fuyou Wang, Ge Chen.
